# Whether high government subsidies reduce the healthcare provision of township healthcare centers in rural China

**DOI:** 10.1186/s12913-021-07201-w

**Published:** 2021-10-30

**Authors:** Chi Shen, Zhongliang Zhou, Sha Lai, Wanyue Dong, Yaxin Zhao, Dan Cao, Dantong Zhao, Yangling Ren, Xiaojing Fan

**Affiliations:** 1 School of Public Policy and Administration, Xi’an Jiaotong University, Xi’an, 710049 China; 2 School of Health Economics and Management, Nanjing University of Chinese Medicine|, Nan Jing, 210023 China; 3 School of Public Health, Health Science Center, Xi’an Jiaotong University, Xi’an, 710061 China

**Keywords:** Incentives, Township healthcare centers, Healthcare reform, China

## Abstract

**Background:**

China’s government launched a large-scale healthcare reform from 2009. One of the main targets of this round reform was to improve the primary health care system. Major reforms for primary healthcare institutions include increasing government investment. However, there are insufficient empirical studies based on large sample to catch long-term effect of increased government subsidy and lack of sufficient incentives on township healthcare centers (THCs), therefore, this study aims to provide additional empirical evidence on the concern by conducting an empirical analysis of THCs in Shaanxi province in China.

**Methods:**

We collected nine years (2009 to 2017) data of THCs from the Health Finance Annual Report System (HFARS) that was acquired from the Health Commission of Shaanxi Province. We applied two-way fixed effect model and continue difference-in-difference (DID) model to estimate the effect of percentage of government subsidy on medical provision.

**Results:**

A clear jump of the average percentage of government subsidy to total revenue of THCs can be found in Shaanxi province in 2011, and the average percentage has been more than 60% after 2011. Continue DID models indicate every 1% percentage of government subsidy to total revenue increase after 2011 resulted in a decrease of 1.1 to 3.5% in THCs healthcare provision (1.9% in medical revenue, 1.2% in outpatient visit, 3.5% in total occupy beds of inpatient, 1.1% in surgery revenue, 2.1% in sickbed utilization rate). The results show that the THCs with high government subsidy reduce the number of medical services after 2011.

**Conclusions:**

We think that it is no doubt that the government should take more responsibility for the financing of primary healthcare institutions, the problem is when government plays a central role in the financing and delivery of primary health care services, more effective incentives should be developed.

**Supplementary Information:**

The online version contains supplementary material available at 10.1186/s12913-021-07201-w.

## Background

In order to deal with the problem of the healthcare system and achieve the goal of establishing a universal coverage healthcare system for all citizens by 2020, the Chinese government launched a large-scale healthcare reform in 2009 [1, 2]. Policies in this round reform can be summarized into five parts: Social health security, Essential medicines, Primary healthcare, Essential public health services program, Public hospitals [3, 4]. To improve the performance of the primary healthcare system is one of the main targets of this round reform. Major reforms for primary healthcare institutions (PHIs) include increasing government investment, eliminating drug mark-ups, and general practitioners contract service [4–6].

Increasing government investment means the government provides financial subsidies to cover all the costs of PHIs, such as construction and equipment expenditure, personnel salary, and operational expenses for public health services [7]. Before the reform, the PHIs’ cost was financed by the government and revenue of PHIs (including drug sales) [8]. According to increasing government investment and eliminating 15% drug mark-ups, China government hopes the PHIs can provide convenient and low-cost essential health services for residents rather than profit-oriented [1].

Along with the change of compensation strategy, the incentives of primary healthcare staff are also changed. After the reform, the total salary of staff in township healthcare centers (THCs) is approved based on the number of staff and service workload, referring to the average salary of the local government staff [7]. The salary consists of basic salary and performance-based bonus. The basic salary shares 60 to 70% of the total salary, performance-based bonus accounts for 30 to 40% of total salary [8]. However, every staff can get 70% of the bonus, only 30% of the bonus is really based on performance [9]. Therefore, the low bonus based on performance leads to a problem - a lack of sufficient incentives.

Due to professional ethics, practice norms, and financial incentives affect the performance of staff in THCs, a lack of sufficient incentives would lead to inefficiencies and poor quality of healthcare services [10]. From 2009 to 2017, the percentage of outpatient services provided by THCs decreased from 16.0 to 13.6%, and inpatients services decreased from 28.7 to 16.6% [11]. One study shows that the compensation and incentive strategy is the key point in the reform of THCs [12]. Another study conducted in Anhui province in China shows that healthcare services provided by THCs with high government subsidy and performance-based salary system have reduced to varying degrees between 2009 and 2010 [9]. In Hubei province in China, the technical efficiency of THCs decreased from 2012, and THCs experienced a decline in productivity [13]. However, existed research have some limitations: small sample (6 and 48 THCs) [9, 13], descriptive analysis, or theoretical analysis [9, 12]**.**

There are insufficient empirical studies based on a large sample to catch the long-term effect of increased government subsidies and lack of sufficient incentives on THCs. This study aims to estimate the effect of the percentage of government subsidy on total revenue on the medical service of THCs. We conducted an empirical analysis by using a balanced panel data that includes 1199 THCs from 2009 to 2017 in Shaanxi province, China.

**Table:** 

**Fig. 1:**
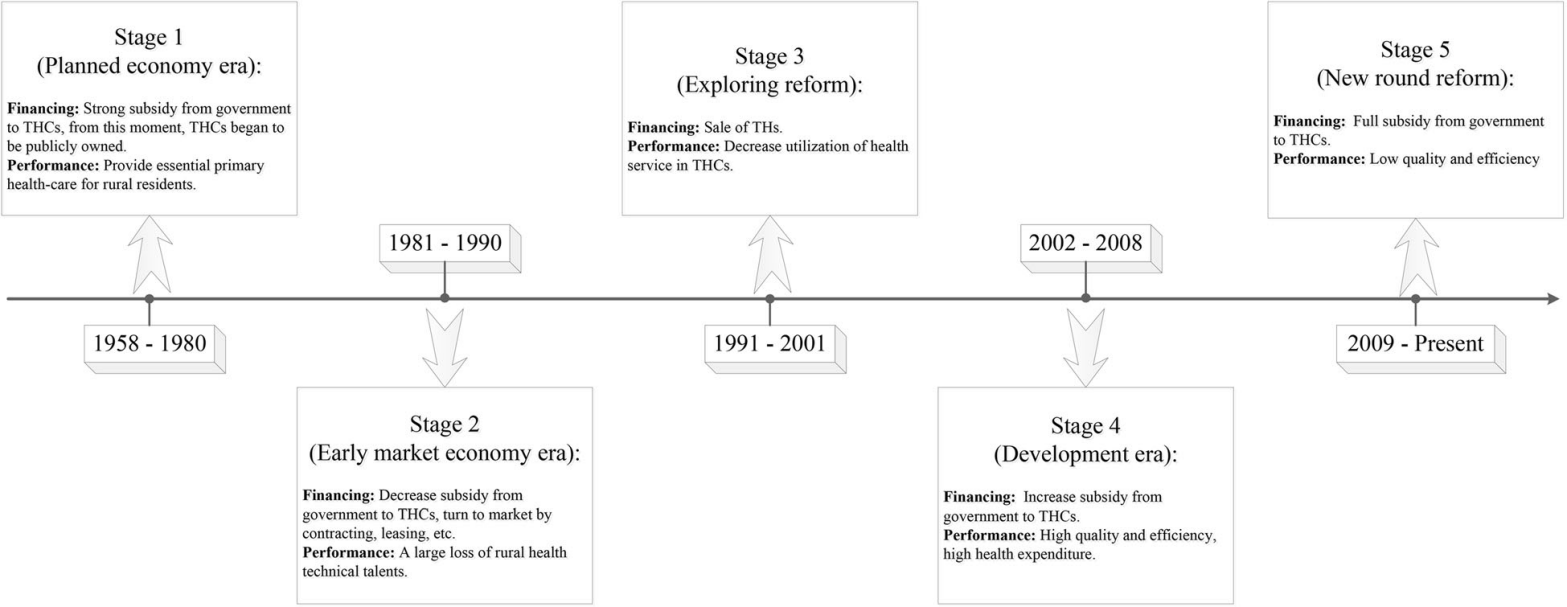
Stages of development of township health centers

**Box 1 The entire history of China’s township healthcare centers**	
The main component of PHIs in rural China is township health centers (THCs). The entire history of China’s THCs is about how to balance the relationship between government and market in terms of financing (Fig. 1). At the stage of the People’s Republic of China funded (1958–1980), the government strongly supported and financed the THCs, and people’s health statue intensely promoted during this period [14]. At the stage of Economic Reform and Open Up (1981–2001), the government changed the financing policy and made the THCs into the market, which caused a massive loss of technical staff from THCs and resident’s healthcare utilization in THCs decreased [14–16]. After aware of the shrinking of THCs, the government began to increase the subsidy to THCs from 2002 and continue to increase it from 2009 [7]

**Table:** 

**Fig. 2:**
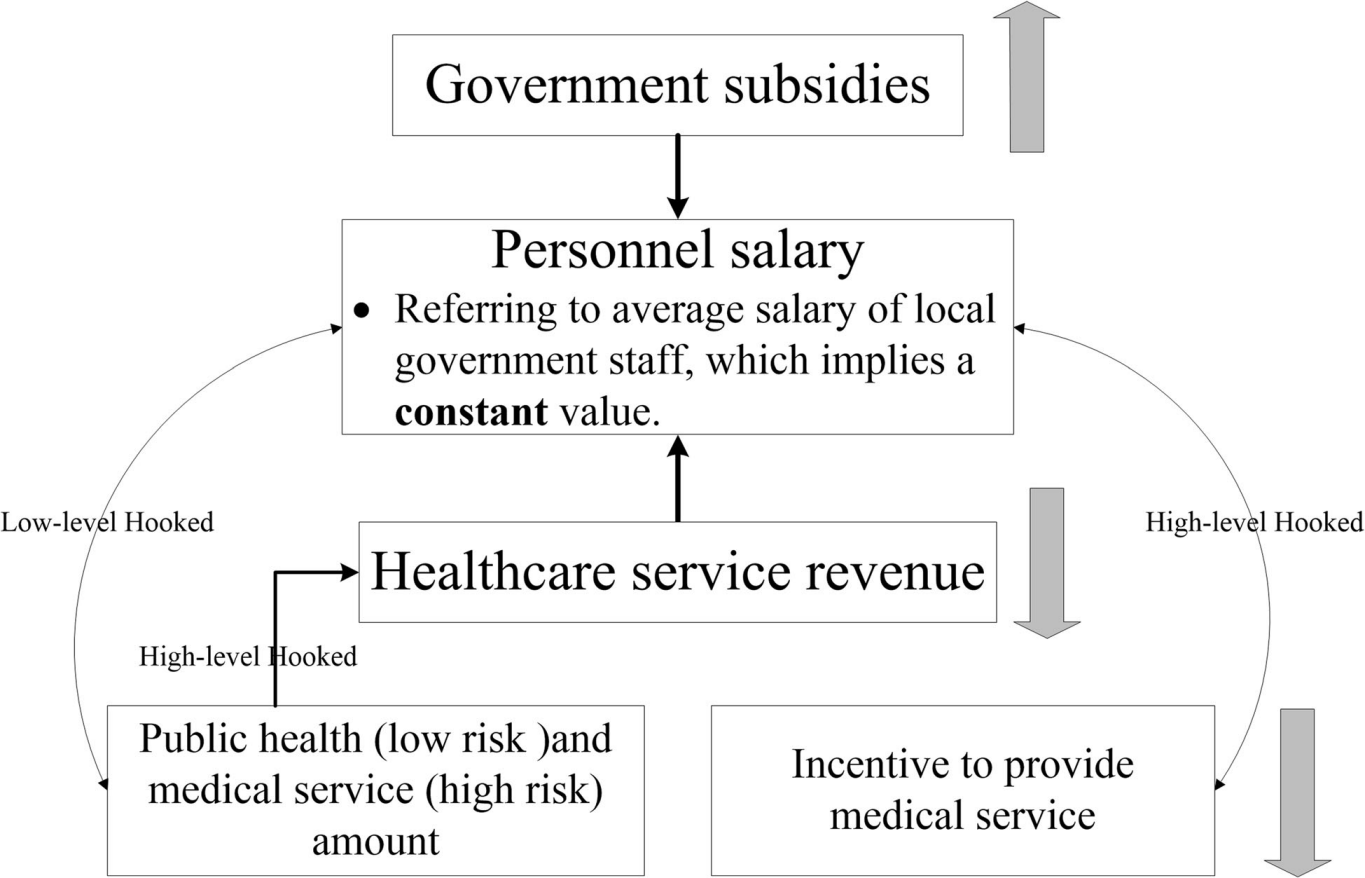
The relationship between government subsidies and incentive to provide medical services in China’s THCs

**Box 2 The relationship between government subsidies and incentive to provide medical services in China’s THCs after 2009**	
Looking at the reform process of THCs in China (Fig. 1), it is easy to see that the government-market pendulum swings back and forth in the healthcare policy sector during the past decades. Along with this swinging, the government subsidies for THCs increased and decreased, the pendulum swung back to the government in the reform of THCs after 2009. In general, government subsidies are not directly tied to employee incentives in the health care sector, but rather have an impact on incentives through employee salaries. In most economic activities, including healthcare, the incentive to provide services is highly hooked to salary. How do the government subsidies affect the incentive through personnel salaries is shown in Fig. 2. Following the increase in government subsidies to THCs, personnel salaries were limited to a relatively fixed level, based on the average salary of local government workers, with the aim of low-level hooking THCs employees’ incomes with healthcare service revenues. This change led to a result where there was not much difference in personnel salaries between employees who did more or less work. Therefore, when the salary was limited to a relatively fixed level, increasing the government subsidies to THCs would make less incentive to provide medical services that are high risk compared to public health services.	

## Methods

### Study site and data collection

Shaanxi province is located in northwest China and has an area of 205,800 km^2^, the total population in 2017 is over 38.35 million [17]. There are 11 prefecture-level cities, 77 counties [18]. The economic and development level of Shaanxi province is the average level of China.

We collected nine years (2009 to 2017) data of THCs from the Health Finance Annual Report System (HFARS) that was acquired from the Health Commission of Shaanxi Province. The HFARS includes resource, service, and finance information of each health institutions hosted by the government. The data include number of medical personnel, number of beds, number of visits in hospitals, and day of in-hospital patient bed occupancy, revenue, medical revenue, and more other provision indicators. Due to changes in administrative divisions and the elimination of missing values, finally, this study included 1199 THCs from total 1552 THCs and constructed a balanced panel dataset [19].

### Indicators and models

We chose the percentage of government subsidy to total revenue of THCs as the explanatory indicator that can identify the degreed of compensation of THCs from the government. Considering the lack of efficient incentives would firstly affect the medical services provided by THCs, we selected the indicators that can reflect the amount of inpatient and outpatient services as the explained variable. Moreover, some confounders will affect the THCs’ provision, such as number of physicians and sickbeds, we chose these confounders as control variables. The detail about variables including in this study can be found in Table 1.

**Table 1:** Detail about indicators in this study

Dependent variables	Control variables	Explanatory variable
Medical revenue	Number of physician	Government subsidy as % of total revenue
Total occupy beds of inpatient	Type of THCs (Center or General)	
Total outpatient visits	Number of authorized bed	
Surgery revenue	Separation between revenue and expenditure	
Sickbed utilization rate		

A two-way fixed-effect model was used to estimate the average treatment effect of the percentage of government subsidy, estimating eq. (1) is written as:

1 $$ {y}_{it}=\beta \cdot {Subsidy}_{it}+{z}_i\delta +\lambda t+{\mu}_i+{\in}_{it} $$

Where,

- where *i* indexes THCs and *t* indexes years.
- *y*_*it*_ is the indicator that can reflect the amount of inpatient and outpatient services of THCs.
- *Subsidy*_*it*_ is the government subsidy as % of total revenue of *i* THCs at *t* time.
- *z*_*i*_ is the control variable.
- *λt* is the year fixed effect.
- *μ*_*i*_ is the THCs fixed effect.
- *ϵ*_*it*_ is the residual error.

According to the official document [20], released on July 6, 2011, government subsidy of THCs increased from 2011 in Shaanxi province. Since there is a clear policy cut-point, we also applied a difference-in-difference (DID) model to compare the average effect of subsidy percentage on the provision of medical services of THCs before and after 2011. However, the DID model used in this study is different from standard DID model [21], the difference is that the intensity of treatment is a continuous measure (i.e., government subsidy as % of total revenue). The detail about the continuous DID model can be found here [22]. Estimating eq. (2) is written as:

2 $$ {y}_{it}=\beta \cdot {Subsidy}_{it}\ast {\mathrm{Year}}^{2011}+{z}_i\delta +\lambda t+{\mu}_i+{\in}_{it} $$

Where,

- where *i* indexes THCs and *t* indexes years.
- *y*_*it*_ is the indicator that can reflect the amount of inpatient and outpatient services of THCs.
- *Subsidy*_*it*_ is the government subsidy as % of total revenue of *i* THCs at *t* time.
- *z*_*i*_ is the control variable.
- *λt* is the year fixed effect.
- *μ*_*i*_ is the THCs fixed effect.
- *ϵ*_*it*_ is the residual error.
- *Year*^2011^ is a dummy variable (2009 and 2010 = 0, after 2011 = 1)

As we found, the distribution of explained variables is over discrete in the process of descriptive analysis, and we made a logarithmic conversion of explained variables in models. All analysis in this study was performed by R 3.5.3 [23].

## Results

### Basic information of explained and explanatory variables

**Fig. 3:**
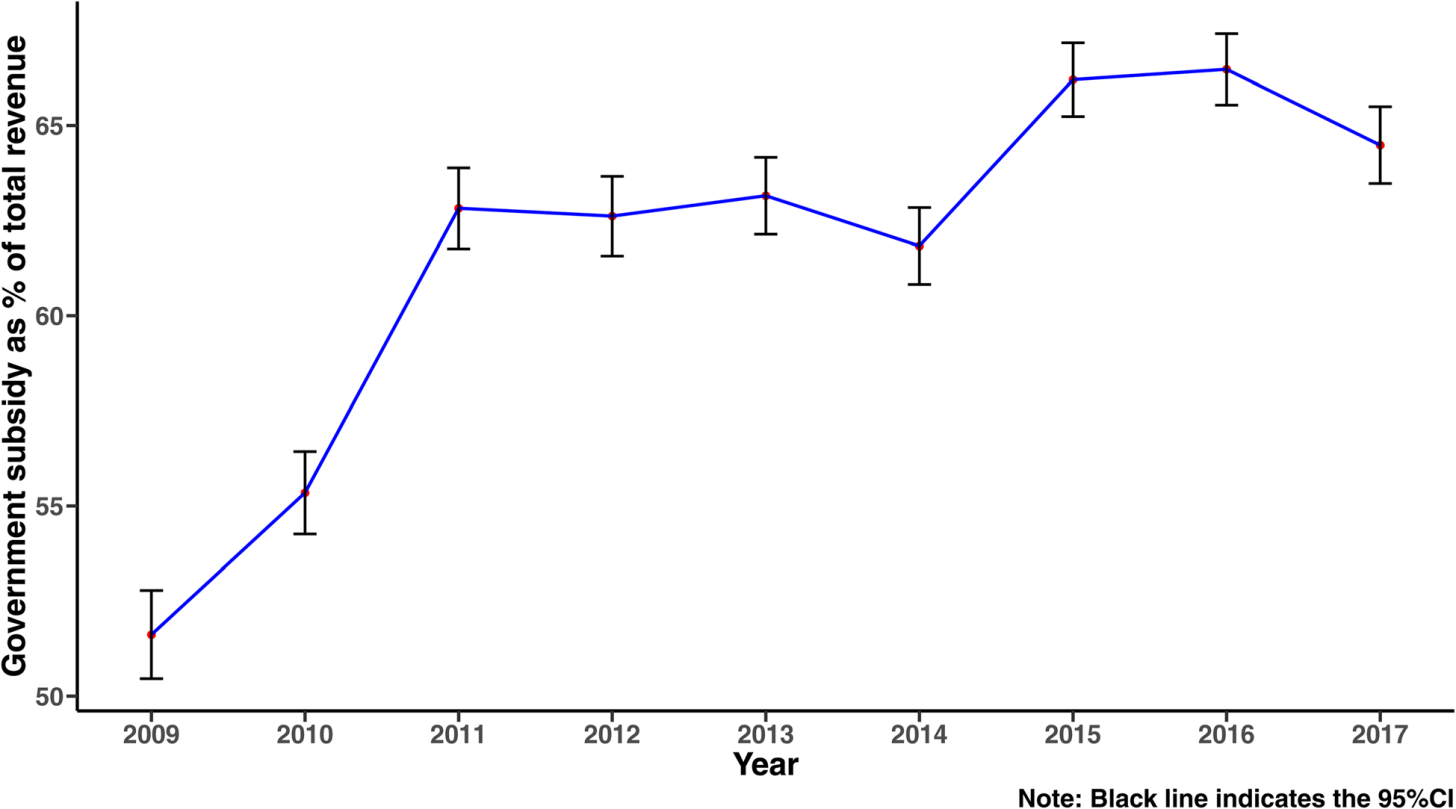
Tread of percentage of subsidy of THCs from 2009 to 2017

**Fig. 4:**
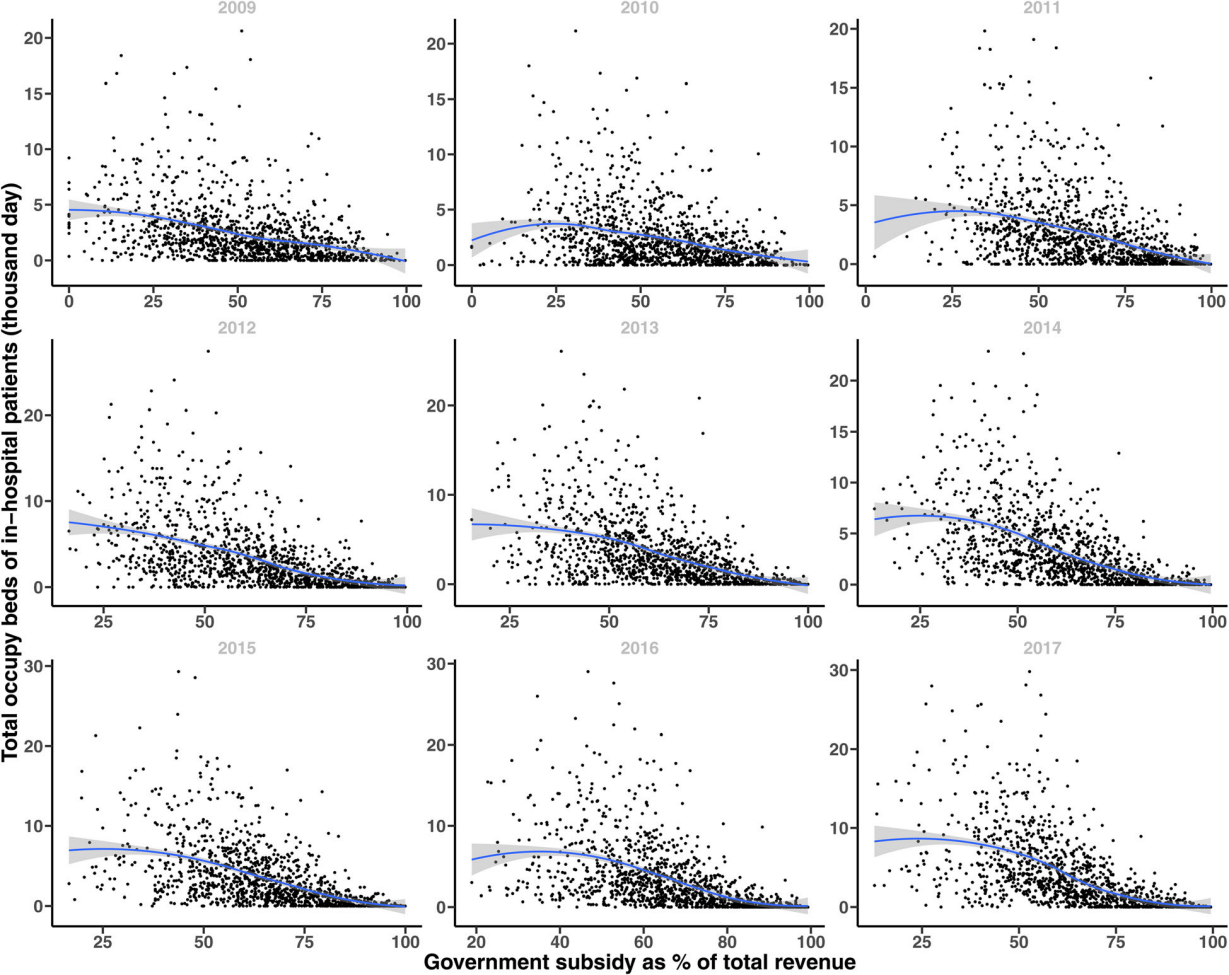
Relationship between subsidy and in-hospital from 2009 to 2017

We can find a clear jump of the average percentage of government subsidy to total revenue of THCs in Shaanxi province in 2011 from Fig. 3, and the average percentage has been more than 60% after 2011. In terms of medical services, medical revenue, and inpatient and outpatient services increased from 2010 to 2017, and there was a decrease in surgical income and no increase in sickbed utilization rate (Table 2). Furthermore, Fig. 4 presents the scatter plots of THCs between government subsidy to total revenue and total occupy beds of inpatient yearly from 2009 to 2017, the locally estimated scatterplot smoothing (LOESS) curves show that the relationship between government subsidy and total occupy beds of inpatient are negative. Other indicators have the same relationship, please see Fig. A1 to Fig. A4 in Additional file).

**Table 2:** Summary of dependent variables

Year	Observation	Medical revenue (RMB)		Outpatient visit		Total occupy beds of inpatient		Surgery revenue (RMB)		Sickbed utilization rate (%)	
		Mean	SD	Mean	SD	Mean	SD	Mean	SD	Mean	SD
2009	1199	234,326.1	317,225.4	11,735.5	11,280.2	2413.9	2673.1	27,241.7	64,269.9	47.0	30.2
2010	1199	260,552.9	354,245.0	11,959.4	11,725.2	2381.2	2759.5	24,353.7	67,400.3	44.3	31.3
2011	1199	746,156.5	790,213.3	12,356.4	12,128.8	2549.2	2984.1	26,435.9	69,750.1	45.9	33.0
2012	1199	971,877.6	1,006,631.0	13,097.8	12,697.1	3253.2	3777.8	27,527.2	75,973.9	49.4	33.5
2013	1199	1,088,195.9	1,073,457.4	13,260.8	12,647.3	3356.3	3767.9	27,798.9	82,212.7	51.0	33.6
2014	1199	1,172,641.7	1,141,956.2	13,598.3	12,306.6	3277.1	3637.1	29,039.5	84,107.3	45.2	32.5
2015	1199	1,258,281.4	1,218,177.7	14,016.6	13,175.1	3342.5	3894.4	26,119.3	78,710.0	44.9	33.0
2016	1199	1,356,923.4	1,312,443.4	14,023.1	12,760.6	3408.1	4196.3	21,359.4	66,077.3	42.2	32.0
2017	1199	1,617,121.6	1,601,525.4	14,498.5	12,989.5	3886.7	4615.6	20,687.7	61,555.1	45.0	33.0

### Two-way fixed effect model result

Based on the results from scatter plots, we used two-way fixed effect models to estimate the average effect of the percentage of government subsidy to total revenue on number of medical services. We performed a Hausman Test to compare fixed effect model with random effect model, the result indicates that fixed effect model is better. Table 3 shows that all the coefficients of the percentage of government subsidy are negative and significant in model 1 to model 5, which indicates that every 1% percentage of government subsidy to total revenue increase resulted in a decrease of 1.3 to 2.6% in THCs healthcare provision (2.4% in medical revenue, 1.3% in outpatient visit, 2.6% in total occupy beds of inpatient, 1.4% in surgery revenue, 1.8% in sickbed utilization rate).

**Table 3:** Result of fixed effect model

	log (medical revenue)	log (outpatient visit)	log (total occupy beds of inpatient)	log (surgery revenue)	log (sickbed utilization rate)
	(1)	(2)	(3)	(4)	(5)
**Subsidy percentage**	− 0.024^***^	− 0.013^***^	− 0.026^***^	− 0.014^***^	− 0.018^***^
	(< 0.001)	(< 0.001)	(0.001)	(0.003)	(0.001)
Type of THCs [C220]	−0.022	− 0.002	0.074	0.071	− 0.038
	(0.036)	(0.038)	(0.109)	(0.226)	(0.068)
Number of Physician	0.005^***^	0.005^***^	0.005^**^	0.011^**^	0.002
	(0.001)	(0.001)	(0.002)	(0.005)	(0.002)
Number of authorized bed	0.006^***^	0.005^***^	0.034^***^	0.018^***^	−0.000
	(0.001)	(0.001)	(0.003)	(0.006)	(0.002)
Separation revenue policy	−0.054^***^	−0.085^***^	− 0.228^***^	−0.290^**^	− 0.108^***^
	(0.020)	(0.022)	(0.061)	(0.128)	(0.039)
Individual effect	YES	YES	YES	YES	YES
Time effect	YES	YES	YES	YES	YES
Observations	10,791	10,791	10,791	10,791	10,791
*R*^*2*^	0.264	0.097	0.063	0.006	0.052
Adjusted *R*^*2*^	0.171	−0.018	−0.055	−0.120	−0.068
*F* Statistic (df = 5; 9579)	688.720^***^	204.784^***^	129.574^***^	10.739^***^	105.410^***^

*Note:* ^***^p < 0.01; ^**^p < 0.05;^*^ *p < 0.1*

*p value of Hausman Test is < 0.05, which indicates that fixed effect model is better*

### [continue difference-in-difference model result

In order to evaluate the average effect of the percentage of government subsidy to total revenue on number of medical services before and after 2011, we applied continue DID model. Table 4 shows that coefficients of interaction between the year dummy variable and the percentage of government subsidy to total revenue are also negative, the coefficients indicate every 1% percentage of government subsidy to total revenue increase after 2011 resulted in a decrease of 1.1 to 3.5% in THCs healthcare provision (1.9% in medical revenue, 1.2% in outpatient visit, 3.5% in total occupy beds of inpatient, 1.1% in surgery revenue, 2.1% in sickbed utilization rate). The results show that the THCs with high government subsidy reduce the number of medical services after 2011.

**Table 4:** Result of continuous measure difference-in-difference model

	log (medical revenue)	log (outpatient visit)	log (total occupy beds of inpatient)	log (surgery revenue)	log (sickbed utilization rate)
	(1)	(2)	(3)	(4)	(5)
Subsidy percentage*Year^**2011**^	−0.019^***^	−0.012^***^	− 0.035^***^	−0.011^***^	− 0.021^***^
	(< 0.001)	(< 0.001)	(0.001)	(0.003)	(0.001)
Type of THCs [C220]	0.020	0.018	0.093	0.096	−0.020
	(0.039)	(0.039)	(0.107)	(0.227)	(0.068)
Number of Physician	0.005^***^	0.005^***^	0.005^*^	0.011^**^	0.002
	(0.001)	(0.001)	(0.002)	(0.005)	(0.002)
Number of authorized bed	0.005^***^	0.004^***^	0.029^***^	0.017^***^	−0.003^*^
	(0.001)	(0.001)	(0.003)	(0.006)	(0.002)
Separation revenue policy	−0.066^***^	−0.090^***^	− 0.227^***^	−0.297^**^	− 0.110^***^
	(0.022)	(0.022)	(0.061)	(0.128)	(0.038)
Individual effect	YES	YES	YES	YES	YES
Time effect	YES	YES	YES	YES	YES
Observations	10,791	10,791	10,791	10,791	10,791
*R*^*2*^	0.151	0.075	0.088	0.004	0.066
Adjusted *R*^*2*^	0.044	−0.042	−0.027	−0.122	−0.052
*F* Statistic (df = 5; 9579)	341.503^***^	154.581^***^	184.786^***^	8.180^***^	135.773^***^

*Note:* ^***^p < 0.01; ^**^p < 0.05;^*^ *p < 0.1*

*p value of Hausman Test is < 0.05, which indicates that fixed model is better*

## Discussion

Our study indicates that, from 2009 to 2017, medical services provided by THCs in Shaanxi province slightly increased, and the government subsidy to THCs experienced substantial growth from 2011. However, the increase of government subsidy did not make the THCs provide more necessary medical services after completing the tasks set by the government. THCs did not meet the health needs of rural residents stimulated by New Rural Cooperative Medical [24, 25].

The findings in our study are similar to the previous studies conducted in Anhui and Hubei province [9, 13], the new contribution of our research is that we confirm the effect of compensation policy changes on the medical services provided by THCs from the perspective of large samples and long-term effects. So, is the incentive method important for primary healthcare institutions? Several review papers indicate that the financing policies for primary health care in China in this round reform might lead to low productivity in primary health-care institutions, and incentive policy is one of the key elements in building an integrated primary health-care system for China [5, 26]. However, it is difficult to make an appropriate incentive strategy. An insurance intervention study conducted in Ningxia province in China revealed that changes in payment method on supply side failed to change residents’ healthcare seeking behavior [27].

The original intention of increasing government subsidy for THCs is to compensate for the shortage of income and expenditure of THCs when eliminating 15% drug mark-ups [7]. Along with the government subsidy increase, the incentive strategy shifts from profit-driven (e.g., prescribing diagnostic tests and drugs) [10] to “approved task, approved revenue and expenditure, performance-based bonus”, which means each THCs will be set an annual task. How to set task goals plays a key role in maintaining the quality and efficiency of health services in THCs. However, factually, the task is simply approved by reference to service population and average health service provision of the THCs over the past three years [20]. Under the situation that high government subsidy, unscientific task setting method, and lack of incentive in salary system, it is common sense that staffs in THCs have no incentives to do extra work after completing the task. Therefore, THCs firstly reduced high-risk medical services, such as hospitalization and surgery, we have reason to believe that THCs with higher government subsidy will reduce medical services compared to THCs with lower government subsidies.

There is a long-standing debate in China that the financing and provision of healthcare services should depend on the market or government. In the past ten years of reform, the Chinese government has invested a large number of funds in the primary healthcare system, but it has not achieved the expected outcomes. It is no doubt that the government should take more responsibility for the financing of primary healthcare institutions. The problem is that when the government plays a central role in the financing and delivery of primary health care services, more effective incentives should be developed. Supply-side incentives can promote to build an integrated healthcare delivery system based on primary healthcare system in China [28]. Fortunately, however, the Chinese government has begun to promote the reform of the salary system of public hospitals, which proposes “two permits”, namely, allowing health institutions to break the limitation of the current level of salary and allowing health institutions to use the revenue balance as personnel reward [29].

There are two limitations in this study. Firstly, as it is difficult to collect the population of towns, we have to use the number of authorized beds of THCs as a proxy variable to control the influence of the population. Secondly, as we mentioned before, services provided by THCs include public health and medical service, however, only indicators of medical service were included in our study because of no suitable indicators about the provision of public health in HFARS.

## Conclusion

Because of the changes in the compensation policy of township health centers and the lack of incentives, the increase of government subsidy did not make the township health centers to provide more necessary medical services, which indirectly leads to a decline in medical service quality and aggravates the problem of “KAN BING NAN”. It is no doubt that the government should take more responsibility for the financing of primary healthcare institutions. The problem is that when the government plays a central role in the financing and delivery of primary health care services, more effective incentives and a more motivating performance appraisal policy should be developed. We suggest that how to trade off the public welfare and efficiency of primary health-care institutions still be a key point in next phase of China’s healthcare reform. Payment methods and some behavioral economics theories (e.g.: loss aversion) can be used to design incentive policies.

## Supplementary Information

**Additional file 1.**

## Data Availability

The datasets used in this study are available from the corresponding author on reasonable request.

## Abbreviations

DID: Difference-in-Difference
HFARS: Health Finance Annual Report System
LOESS: Locally Estimated Scatterplot Smoothing
PHIs: Primary Healthcare Institutions
THCs: Township Healthcare Centers

## References

[1] Central Committee of the Communist Party of China and State Council (2009). Opinions on Deepening Health System Reform.

[2] McCollum R, Chen L, ChenXiang T, Liu X, Starfield B, Jinhuan Z, Tolhurst R (2014). Experiences with primary healthcare in Fuzhou, urban China, in the context of health sector reform: a mixed methods study: Experiences with primary healthcare in Fuzhou, China. Int J Health Plann Mgmt.

[3] Guo Z, Guan X, Shi L (2017). The impacts of implementation of National Essential Medicines Policies on primary healthcare institutions: a cross-sectional study in China. BMC Health Serv Res.

[4] Meng Q, Mills A, Wang L, Han Q (2019). What can we learn from China’s health system reform?. BMJ.

[5] Li X, Lu J, Hu S, Cheng K, De Maeseneer J, Meng Q (2017). The primary health-care system in China. Lancet.

[6] Yip W, Fu H, Chen AT, Zhai T, Jian W, Xu R, Pan J, Hu M, Zhou Z, Chen Q, Mao W, Sun Q, Chen W (2019). 10 years of health-care reform in China: progress and gaps in universal health coverage. Lancet.

[7] General Office of the State Council of China (2010). Opinions on Establishment and Optimization of the Reimbursement Scheme for Primary Health Care Facilities.

[8] Ma X, Wang H, Yang L, Shi L, Liu X (2019). Realigning the incentive system for China’s primary healthcare providers. BMJ.

[9] He P, Sun Q, Liu B, Zuo G, Xue Y, Li K (2013). The change of compensation and incentive policy affecting the income of primary care providers and health care delivery of township health center. Chin Health Serv Manage (In Chinese).

[10] Yip WC-M, Hsiao W, Meng Q, Chen W, Sun X (2010). Realignment of incentives for health-care providers in China. Lancet.

[11] National Health Commission of the People’ s Republic of China (2018). China health statistical yearbook 2018.

[12] Xu J (2010). Compensation mechanism of township health center in China. Health Econ Res (In Chinese).

[13] Cheng Z, Cai M, Tao H, He Z, Lin X, Lin H, Zuo Y (2016). Efficiency and productivity measurement of rural township hospitals in China: a bootstrapping data envelopment analysis. BMJ Open.

[14] Cheng J, Yuan Y, Lu W, Yang L (2017). Primary health care in China: is China’s health reform reform for the whole nation?. Prim Health Care Res Dev.

[15] Liu X, Xu L, Wang S (1996). Reforming China’s 50 000 township hospitals—effectiveness, challenges and opportunities. Health Policy.

[16] Wang W, Yu Y, Wang Z (2011). Empirical study on the history of rural hospitals in China. J Anhui Agric Sci (In Chinese).

[17] Shaanxi Provincial Bureau of Statistics (2018). Shaanxi Statistical Yearbook 2018, 4-1: Population and Its Composition.

[18] Shaanxi Provincial Bureau of Statistics (2018). Shaanxi Statistical Yearbook 2018, 1-1: Divisions of Administration Areas in Shaanxi.

[19] Shaanxi Provincial Bureau of Statistics (2018). Shaanxi Statistical Yearbook 2018, 19-3: number of various health units, Beds and Staff.

[20] Shaanxi Provincial People’s Government (2011). Opinions on deepening the comprehensive reform of primary health care institutions of the Shaanxi provincial People’s government.

[21] Ashenfelter O, Card D (1984). Using the longitudinal structure of earnings to estimate the effect of training programs.

[22] Nunn N, Qian N (2011). The Potato’s contribution to population and urbanization: evidence from a historical experiment. Q J Econ.

[23] R Core Team (2020). R: A language and environment for statistical computing. Vienna, Austria.

[24] Yu B, Meng Q, Collins C, Tolhurst R, Tang S, Yan F, Bogg L, Liu X (2010). How does the new cooperative medical scheme influence health service utilization? A study in two provinces in rural China. BMC Health Serv Res.

[25] Zhang D, Shi L, Tian F, Zhang L (2016). Care utilization with China’s new rural cooperative medical scheme: updated evidence from the China health and retirement longitudinal study 2011–2012. IntJ Behav Med.

[26] Yip W, Hsiao W (2014). Harnessing the privatisation of China’s fragmented health-care delivery. Lancet.

[27] Powell-Jackson T, Yip WC-M, Han W (2015). Realigning demand and supply side incentives to improve primary health care seeking in rural China: Improving primary health care seeking in rural China. Health Econ.

[28] Shen M, He W, Li L (2020). Incentives to use primary care and their impact on healthcare utilization: evidence using a public health insurance dataset in China. Soc Sci Med.

[29] Ministry of Human Resources and Social Security of China (2017). Notice on Expanding the Pilot Reform of the Salary System in Public Hospitals.

