# Supplementary material for: Whether high government subsidies reduce the healthcare provision of township healthcare centers in rural China
Source: BMC Health Serv Res. 2021 Oct 30;21:1184. doi: 10.1186/s12913-021-07201-w (PMC8557613; doi:10.1186/s12913-021-07201-w)
Supplement: Supplementary file 1 — Additional file 1. [file 12913_2021_7201_MOESM1_ESM.docx]

**Additional file.1**


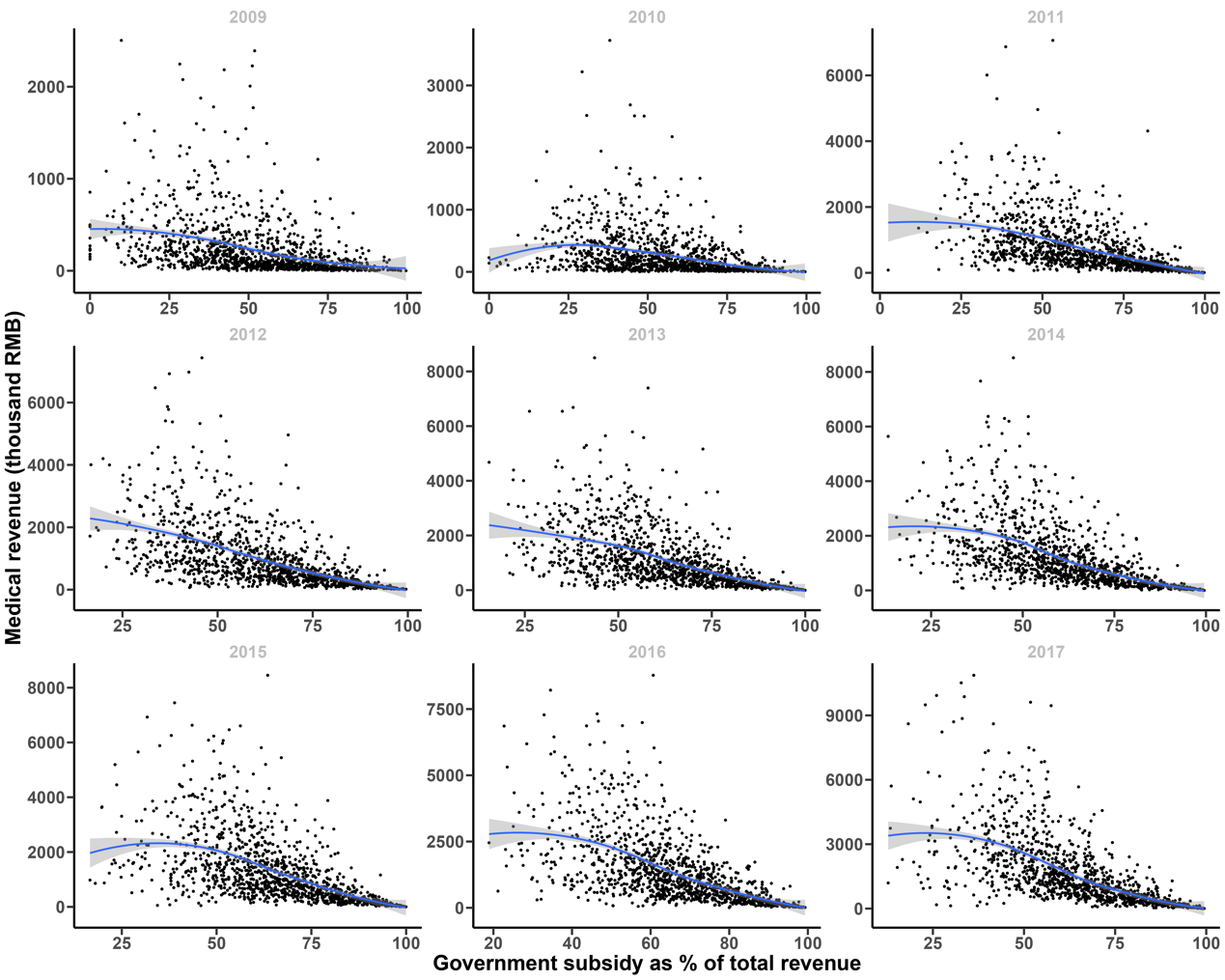


Figure.A1 Relationship between subsidy and medical revenue from 2009 to 2017


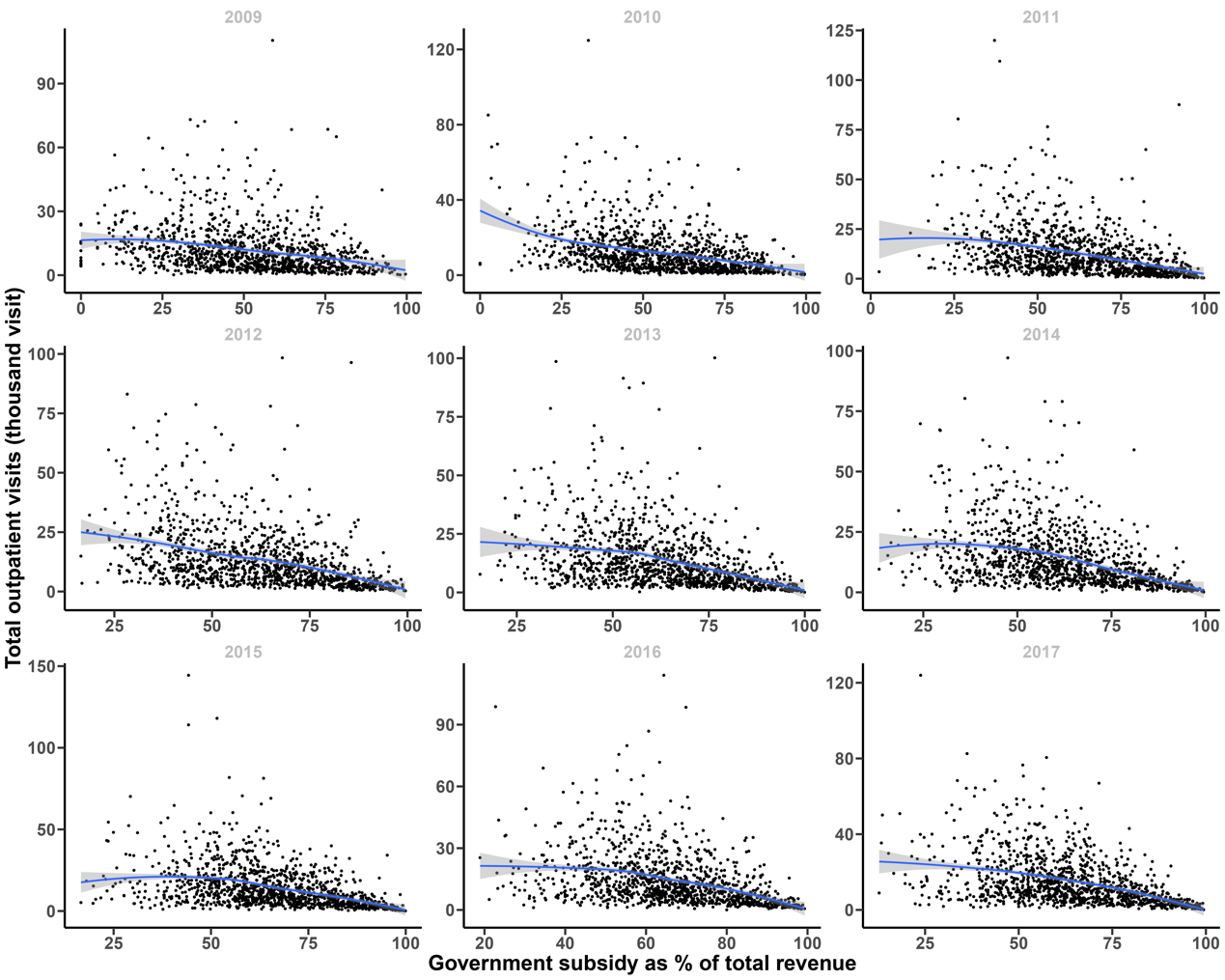


Figure.A2 Relationship between subsidy and outpatient visit from 2009 to 2017


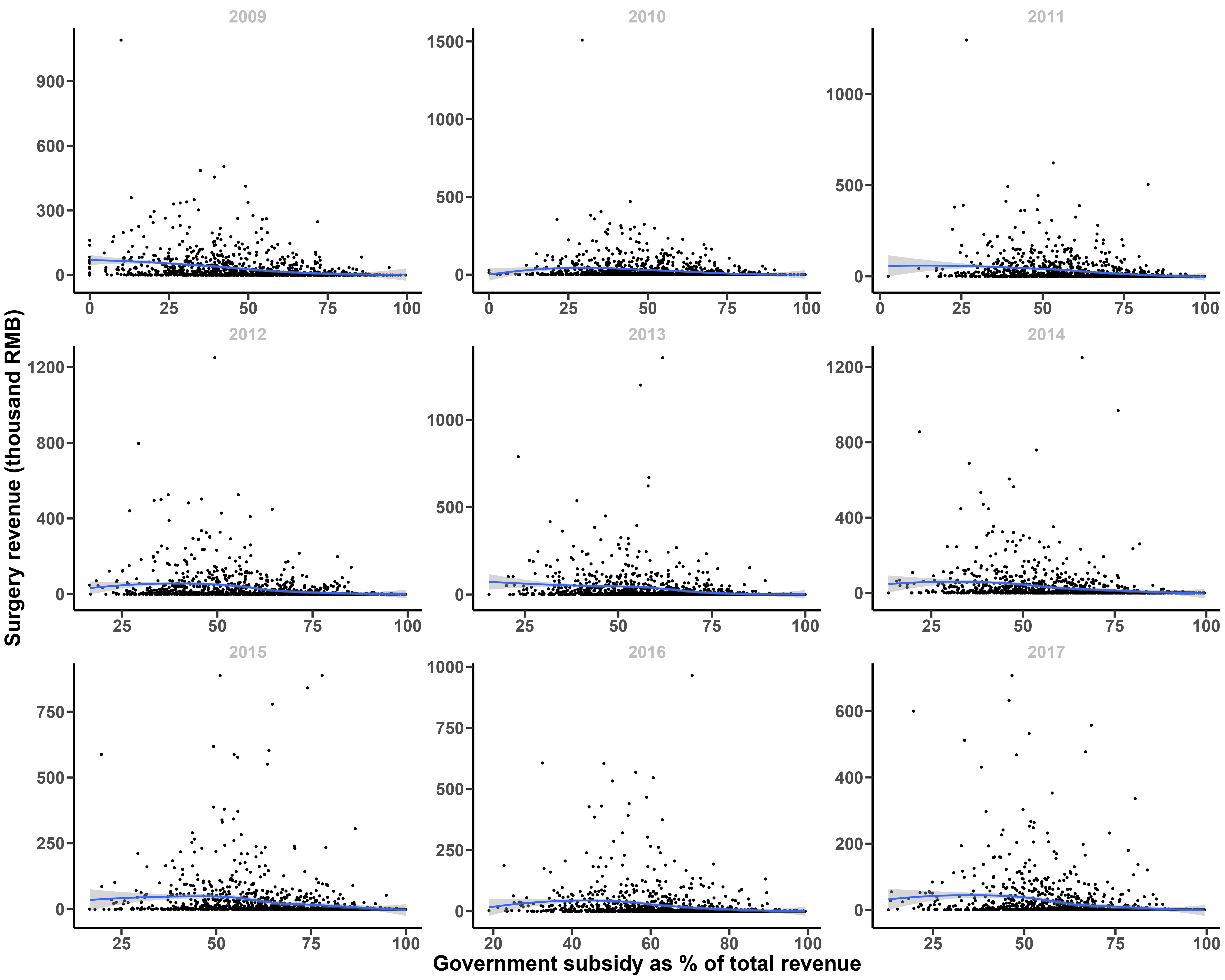


Figure.A3 Relationship between subsidy and surgery revenue from 2009 to 2017


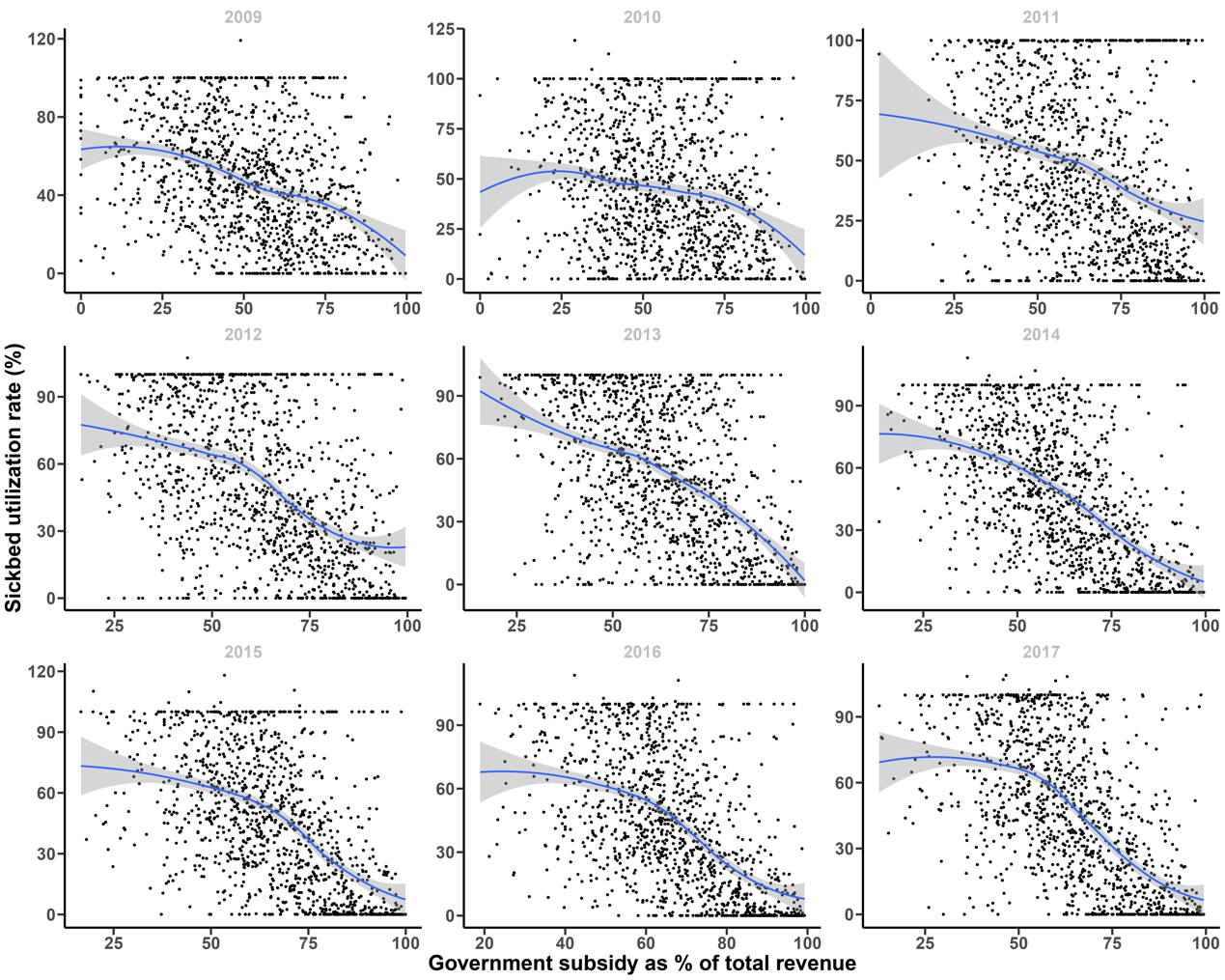


Figure.A4 Relationship between subsidy and sickbed utilization rate from 2009 to 2017
